# The Use of a Predictive Habitat Model and a Fuzzy Logic Approach for Marine Management and Planning

**DOI:** 10.1371/journal.pone.0076430

**Published:** 2013-10-11

**Authors:** Tarek Hattab, Frida Ben Rais Lasram, Camille Albouy, Chérif Sammari, Mohamed Salah Romdhane, Philippe Cury, Fabien Leprieur, François Le Loc’h

**Affiliations:** 1 UR 03AGRO1 Ecosystèmes et Ressources Aquatiques, INAT (Institut National Agronomique de Tunisie), Tunis, Tunisia; 2 UMR 212 Ecosystèmes Marins Exploités, IRD (Institut de Recherche pour le Développement), Sète, France; 3 Département de biologie, Chimie et Géographie, UQAR (Université du Québec à Rimouski), Québec, Canada; 4 Laboratoire du milieu marin, INSTM (Institut National des Sciences et Technologies de la Mer), Salammbô, Tunisia; 5 Laboratoire Ecologie des Systèmes Marins Côtiers UMR 5119, UM2 (Université de Montpellier 2), Montpellier, France; Macquarie University, Australia

## Abstract

Bottom trawl survey data are commonly used as a sampling technique to assess the spatial distribution of commercial species. However, this sampling technique does not always correctly detect a species even when it is present, and this can create significant limitations when fitting species distribution models. In this study, we aim to test the relevance of a mixed methodological approach that combines presence-only and presence-absence distribution models. We illustrate this approach using bottom trawl survey data to model the spatial distributions of 27 commercially targeted marine species. We use an environmentally- and geographically-weighted method to simulate pseudo-absence data. The species distributions are modelled using regression kriging, a technique that explicitly incorporates spatial dependence into predictions. Model outputs are then used to identify areas that met the conservation targets for the deployment of artificial anti-trawling reefs. To achieve this, we propose the use of a fuzzy logic framework that accounts for the uncertainty associated with different model predictions. For each species, the predictive accuracy of the model is classified as ‘high’. A better result is observed when a large number of occurrences are used to develop the model. The map resulting from the fuzzy overlay shows that three main areas have a high level of agreement with the conservation criteria. These results align with expert opinion, confirming the relevance of the proposed methodology in this study.

## Introduction

Understanding species distributions is essential for conservation planning and forecasting [1], particularly in the present context of stock depletion and species extinction. Species distribution maps play an important role in developing spatial management measures such as the identification of Essential Fish Habitats or the establishment Marine Protected Areas (MPAs) [2], [3], which in turn contribute to sustainable ecosystem-based marine management [3]–[5]. However, information on the true distribution of many marine organisms remains limited, particularly for species that are difficult to detect [6]. Modelling species distributions based on data samples is one solution to address this lack of knowledge. For instance, Species Distribution Models (SDMs) relate species’ distributions based on data samples with the associated environmental and geographical characteristics of the surveyed locations [7].

Nevertheless, the perception remains that the distributions of marine species are uncertain and dependent on the sampling process used to generate the models. Bottom trawls are commonly used as a sampling technique to assess the spatial distribution of commercial species and to obtain fisheries-independent abundance data [8]. However, there are some limitations in the species detections associated with this technique due to a range of factors, e.g., catchability, gear efficiency, and gear-specific selectivity [8]. A common problem in recording species’ distribution results from a false absence, which occurs when a species is not available for capture despite occupying the site, or when a species occurs at a site but is simply not captured. Such data can severely limit the fit of many SDMs [9] and can decrease the reliability of prediction models (see [10], [11]). The issue of false absences is further complicated by locations with favourable environmental conditions but where species are absent due to biotic interactions, dispersal limitations or fishing pressure. This latter case is particularly critical when modelling commercial exploited species [12]. Yet the problem of imperfect detection when modelling marine species distributions has been rarely mentioned in the literature [13].

Confirmed absences are very difficult to obtain, especially from bottom trawl survey data and for mobile species. A much higher level of sampling effort is required to ensure their reliability relative to presence data [14]. To cope with the lack of confirmed absence data, presence-only models, or profile techniques, have often been used [12]. These models differ from the group discrimination approaches that require presence–absence or abundance data [15]. Some well-known examples of profile-type models are the Ecological Niche Factor Analysis (ENFA; [16]), the Genetic Algorithm for Rule-Set Prediction [17], and the maximum entropy method [18]. Comparisons between the various SDMs reveal that group discrimination approaches tend to perform better than the profile-type, or presence-only models [19]–[21]. A common problem of the profile-type models is that their predictions are often overly optimistic, i.e., they predict the species occurring at too many locations [22].

In order to use group discrimination approaches, artificial absence data are increasingly being used in situations where no confirmed absence data are available. Typically called pseudo-absences, artificial absences are generated and inserted into the selected model in lieu of confirmed absence data. The method selected to generate the pseudo-absences is particularly important because it can influence the final quality of the model [21], [22]. Several approaches have been suggested for the generation of pseudo-absences: (*i)* a random selection of absence points across the entire available area (e.g., [21], [23]; (*ii*) a random selection with a geographically-weighted exclusion (e.g., [24]; and (*iii)* a selection of sampled locations (i.e., occurrence locations for other species) at which the target species has not been recorded (e.g., [5]). However, there are several issues with these approaches. The first two may produce false absences, even in environmentally-favourable areas for the species [25], [26]. The latter approach is unsuitable for bottom trawl survey data because it is unlikely that an entire target group of species caught by a particular type of fishing gear will share a similar sampling bias.

To deal with presence-only records, Engler *et al*. [22] suggested an intermediate methodology between presence-only and presence-absence distribution models. This approach proposed the use of a habitat suitability map as a way of selecting weighted pseudo-absence data points. These points are added to the original presence-only data and used to improve the logistic regression procedure. This mixed method could be more suitable for bottom trawl survey data than the other approaches outlined above.

Distribution models often result in a predicted probability surface that is then translated into a presence-absence classification map for use in different conservation applications [27]. For instance, these presence-absence maps are commonly aggregated (the ‘predict first, assemble later’ strategy from [28]) to identify areas that will experience the greatest changes in species composition due to climate change [29]. To convert a probability surface into a binary map, a number of threshold selection methods have been proposed [27]. Given the variety of approaches available to generate a dataset, the method chosen can have a dramatic effect on a model’s accuracy and its predictions [27], [30], as well as the subsequent conservation planning decisions and outcomes [31]. To address the uncertainty in the presence-absence classification map, we propose the use of a fuzzy logic approach that can be applied in situations where vagueness and uncertainty exist. The fuzzy logic approach has several advantages in situations where: *(i)* there are no clear cut definitions; and *(ii)* results cannot be categorised as either 0 or 1 [32]. Given its current application in a range of scientific disciplines [33], it is logical to extend the use of fuzzy logic to the threshold selection procedure in the context of SDMs.

Predicting the distributions of commercially targeted marine species is particularly urgent in over-exploited and damaged ecosystems, such as the Gulf of Gabes. With a soft bottom, shallow slope and a high diversity of fishes, the Gulf of Gabes is the most important fishing ground in Tunisia [34], [35]. This coastal area supports 60% of the national fishing fleet and contributes 42% of the national annual fish and crustacean production [36]. This intense fishing activity began in the early 1980s, after which fisheries experienced large fluctuations in landings until the late 1990s when catches began to progressively decline [35]. This was mainly due to the decreasing of demersal stocks caused by intense bottom trawling activities. Depletion rates are so alarming that in the near future the Gulf of Gabes will be subject to a habitat conservation management plan that excludes trawling activities.

In this study, we initially assess the reliability of the mixed approach proposed by Hengl *et al*. [37]. We combine ENFA predictions for generating pseudo-absences and regression-kriging (RK) for modelling the spatial distribution of 27 commercially-targeted species in the Gulf of Gabes based on bottom trawls survey data. We then propose a fuzzy logic framework to transform modelled probabilities of occurrence into binary predictions of species presence and absence. We illustrate this framework by applying it to the task of identifying areas that meet the conservation targets for the deployment of artificial anti-trawling reefs (AARs) in the Gulf of Gabes.

## Materials and Methods

### Study Area

The Gulf of Gabes is located in the southern Mediterranean Sea and covers the second widest continental shelf area of this semi-enclosed sea. The Gulf of Gabes has high fisheries productivity and it serves as a feeding and reproduction area for numerous populations of fishes and crustaceans [34]. Indeed, this ecosystem supports one of the most extensive biocenosis of seagrass (*Posidonia oceanica*; [38]), which constitutes a major nursery site for several marine species [39]. Accordingly, the Gulf of Gabes is one of the most productive ecosystems in the Mediterranean Sea and has great economic and ecological importance [38].

Bottom trawling is the predominant fishing activity in this area and the gear type that has the largest impact on the target demersal fishes [34]. The regular incursions of trawlers into areas that are shallower than their regulated depth have led to the extensive degradation of *P. oceanica* meadows [40]. Due to the lack of monitoring and surveillance activities carried out by the marine police and fishery authorities, illegal fishing still takes place. Consequently, effective management measures are required to prevent illegal fishing activities.

The proposed fisheries management plan for the Gulf of Gabes includes a perimeter of AARs that combine anti-trawling structures with artificial reefs. The major functions of these structures will be to protect the coastal zone marine ecosystems and species from the mechanical impacts of trawling. This measure will be especially important for high diversity communities, such as the *P. oceanica* seagrass beds and associated fauna of biological interest [41]. In addition, AARs aim to reduce the fishing mortality of commercial species, to protect nursery areas and juvenile fish, to create new fishing grounds and/or improve existing grounds, and to increase natural productivity [42]. The AARs will be deployed in areas that are chosen by considering both the presence of favourable habitats of commercial species and areas of high-density *P. oceanica* seagrass beds.

### Bottom Trawl Survey Data and Model Variables

**Figure 1:**
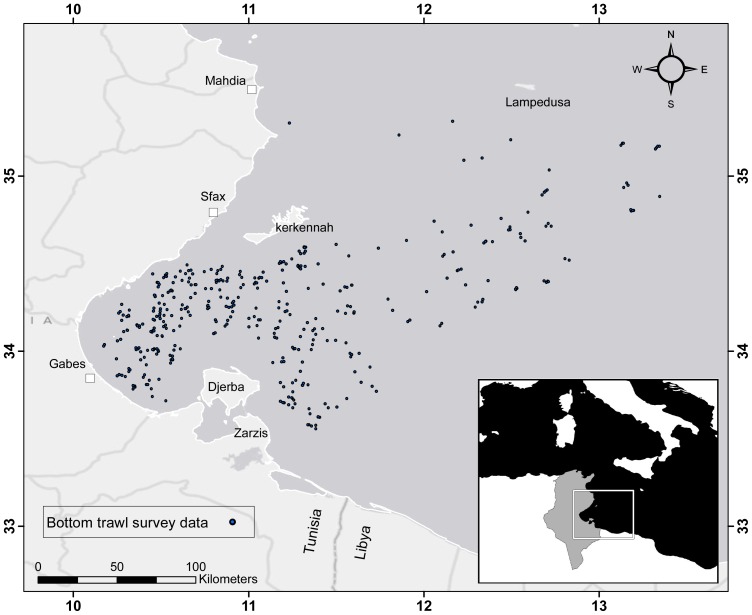
The Gulf of Gabes study area with black points indicating the location of the benthic trawls used in this study.

Species occurrence data were collected from the Tunisian bottom trawl survey database gathered by the National Institute of Marine Sciences and Technologies (INSTM, Tunisia) onboard the R/V Hannibal. The sampling net used (vertical opening trawl, GOV: 42/55) had a 20 mm diamond stretched mesh at the cod end and a 15 m horizontal opening. The trawl was towed at a speed of 2.9 knots for one hour and sampling areas were fixed according to a stratified random sampling based on three depth strata (0–25 m, 25–65 m, and 65–200 m). A total of 360 trawl hauls were completed between 1998 and 2005. Central point geo-referenced position data for each trawl haul and the associated catch contents were extracted from the database (Figure 1).

**Figure 2:**
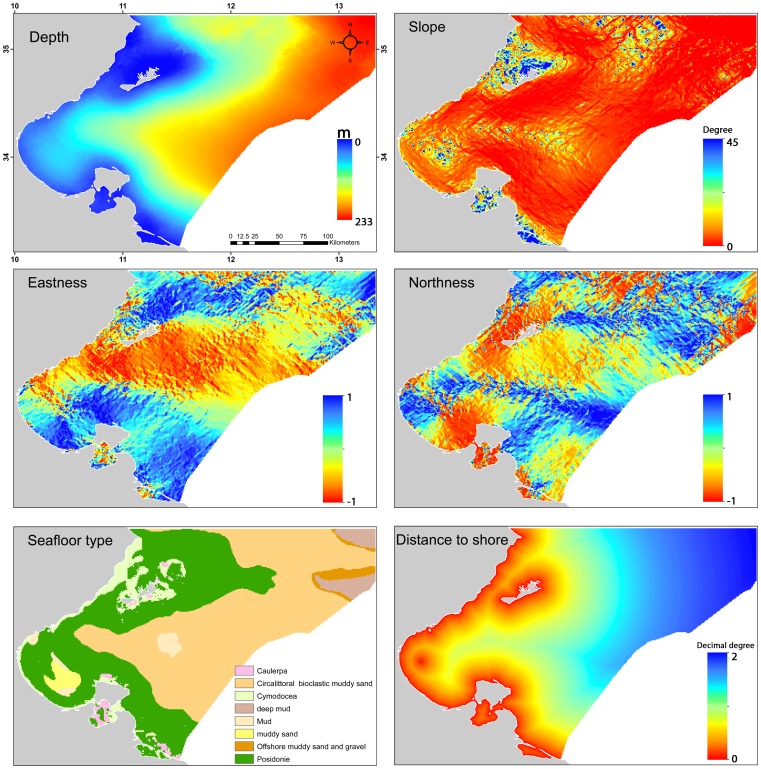
Maps of the six habitat variables (including two derived variables) used to generate the predicted distribution maps.

To build the SDMs, we selected four local habitat variables (depth, slope, aspect, and seafloor type) and one spatial predictor (distance to shore). Previous studies undertaken at a similar spatial extent showed that these variables had a strong influence on species distributions in coastal environments (Figure 2) (e.g., slope [43], [44], depth [5], [45], aspect [43], [44], [46], distance to shore [47], [48], and seafloor type [5]).

The bathymetry of the Gulf of Gabes was extracted as a digital gridded depth data set from a digital elevation model with a 90 m resolution. The bathymetric elevation data was derived from source soundings collected by the INSTM and referenced to the local tidal datum. The seafloor bathymetric slope and aspect were derived from the bathymetric base map using a 3×3 cell neighbourhood window around the processing cell. Respectively, these represent the rate of change in bathymetry and the azimuthal direction of the steepest slope over the analysis window. The aspect was transformed into two derived variables: Eastness (values close to 1 represent an eastward aspect, while values close to –1 represent a westward aspect) and Northness (values close to 1 represent a northward aspect, while values close to –1 represent a southward aspect).

To develop the map of the distance to shore, Euclidean distances were calculated from the shorelines and islands throughout the study area based on a gridded map with a 90 m resolution. Release and recapture locations were then sampled using ArcGIS 10 to obtain distance values. Digital seafloor type data were obtained from the INSTM. The original 15 seafloor types were grouped into eight broader seafloor types that represent the relatively distinct physical environments thought to influence the distributions of demersal marine species. We mapped the seafloor type by attributing a seafloor category to the center of each 0.0081 km^2^ grid cell.

### Selected Species

Since the advent of bottom trawl surveys in the Gulf of Gabes, 152 different species of fishes, cephalopods, and crustaceans have been identified. As small sample sizes pose challenges to any statistical analyses and result in decreased predictive potential, we decided to concentrate on the relatively common species (defined here as species present in >10% of trawl hauls). Having applied this criterion we retained a total of 27 species: 20 fishes, four cephalopods, two decapods, and one stomatopod (Table 1). Collectively, these species constituted 60% of total biomass of the landings and 98% of bottom trawl landings in the Gulf of Gabes [36].

**Table 1:** Code, scientific name and the number of occurrences of each of the 27 species modelled in this study.

Code	Scientific name	Number of occurrences	Code	Scientific name	Number of occurrences
BALICAR	*Balistes carolinensi*	87	OCTOVUL	*Octopus vulgaris*	77
BOOPBOO	*Boops boops*	299	PAGEERY	*Pagellus erythrinus*	223
DIPLANN	*Diplodus annularis*	190	PAGRCAE	*Pagrus caeruleosticutus*	85
ELEDMOS	*Eledone moschata*	64	PENAKER	*Penaeus kerathurus*	219
ENGRENC	*Engraulis encrasicolus*	47	POMTSAL	*Pomatomus saltator*	51
GOBINIG	*Gobius niger*	61	SCOMSCO	*Scomber scombrus*	95
LITHMOR	*Lithognathus mormyrus*	111	SEPIOFF	*Sepia officinalis*	270
LOLIVUL	*Loligo vulgaris*	134	SERAHEP	*Serranus hepatus*	34
MERLMER	*Merluccius merluccius*	164	SOLEAEG	*Solea aegyptica*	104
METAMON	*Metapenaus monoceros*	46	SPARAUR	*Sparus aurata*	34
MUGICEP	*Mugil cephalus*	38	SPICMAE	*Spicara maena*	62
MULLBAR	*Mullus barbatus*	191	SQUIMAN	*Squilla mantis*	43
MULLSUR	*Mullus surmuletus*	69	TRACTRA	*Trachurus trachurus*	169
MUSTMUS	*Mustelus mustelus*	62			

### The Modelling Framework

**Figure 3:**
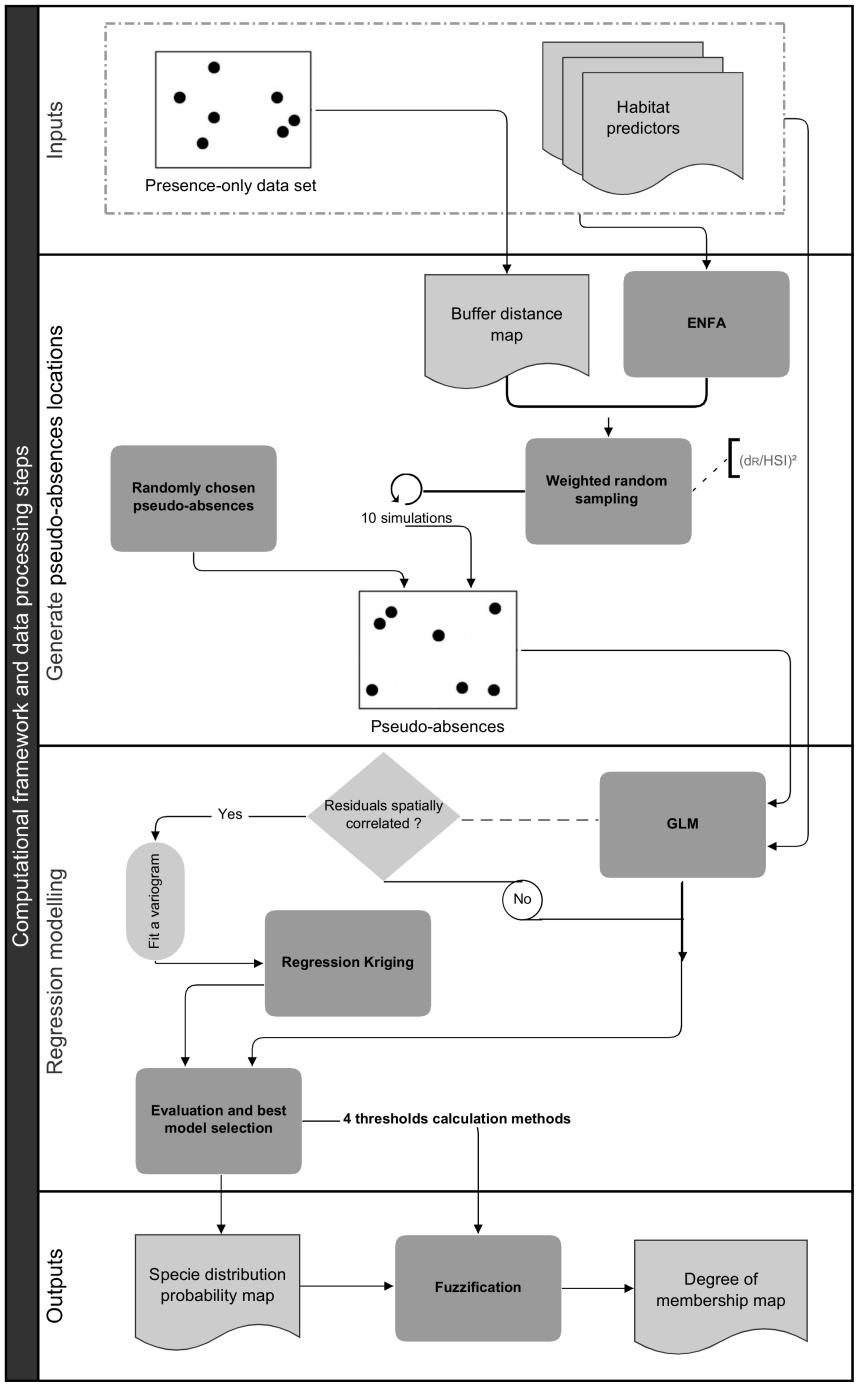
Computational framework and data processing steps.

Confirmed absence data were not available for the 27 species selected for this study. As group discrimination approaches usually perform better than presence-only models (as discussed above), we selected a hybrid approach. This approach combines group discrimination and profile techniques to model species distributions (Figure 3) [22], [25], [37], [49].

#### Presence-only models

ENFA [16] was used to create an habitat suitability map that depicts areas where species are unlikely to occur. ENFA is a specific ordination technique that compares a species’ environmental niche and the environmental characteristics of the study area and assigns a degree of suitability to each point on a map (typically from 0 to 100). Thus, it quantifies the dissimilarity between an ecological niche and the ecological space. The first component of the technique, called the marginality factor, is defined as the standardised difference between the centroids of the ecological space and the ecological niche. The second component, the specialisation factors, are successively extracted from the n–1 residual dimensions and represent the narrowness of the ecological niche relative to the ecological space [16]. ENFA was preferred to another widely used presence-only model, namely maximum entropy method, as several studies recently showed that this technique may lead to spurious inferences (e.g., [50]). Then, ENFA is recognized as one of the best presence-only methods to model habitat suitability for marine species (see [6], [51]).

To evaluate the accuracy of the ENFA, we performed a Monte-Carlo randomisation test with 100 permutations. This test for the significance of the marginality factor by randomising the locations of selected species within the study area. At each permutation the ENFA was performed on the random locations and the results were then evaluated against the observed locations. We ran the ENFA using the *R* adehabitat package [52], [53].

#### Simulation of pseudo-absences

To generate pseudo-absence data, two methods were used to choose geographic coordinates: (*i*) at random across the Gulf of Gabes; and (*ii*) weighted by ENFA predictions and the geographical location of presence-only records [37]. We undertook both methods with the aim of assessing the level of improvement gained by using the environmentally- and geographically-weighted method as compared with the random method. The weighted method proposed by Hengl *et al.* [37] is based on both the Habitat Suitability Index (HSI, derived through ENFA) and the distance from the observations that are subsequently used to weight pseudo-absence points (Figure 3). Given that HSI values are scaled between 0 and 100, Hengl *et al.* [37] defined the probability distribution (τ) used to generate the pseudo-absence locations as:
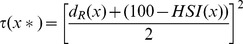
(1)where d_R_ is the normalised distance in the range [0, 100%], i.e., the distance from the observation points divided by the maximum distance. The square term is used to ensure that there are progressively more pseudo-absences at the edge of low HSI and large distances will approximately follow a Poisson distribution. In this way, pseudo-absences are located both in areas of low HSI (unsuitable habitat) and further away from the occurrence locations.

Based on Equation 1, the HSI and the map with buffers around the occurrences were combined to create a weighted map. We then performed the random generation of points with a probability density proportional to the values of the weighted map. To account for the variability arising from this weighted selection, 10 groups of pseudo-absences were generated. This allowed us to assess the stability of the final predictions for different simulations. For each set, the number of simulated pseudo-absences was equal to the number of presences. This is supported by the statistical theory of model-based designs, also known as “D-designs” [37]. According to this theory, the optimal design to minimise prediction variance is when an equal number of observations are at opposite value extremes [37], [54] and there is a higher spreading in the feature space.

A total of 11 groups of pseudo-absences were obtained. One group was generated entirely at random and the remaining 10 groups were weighted by ENFA predictions and the geographical locations of the presence-only records (Figure 3).

#### Regression modelling

Once the pseudo-absences were simulated, they could then be combined with the occurrence locations to build a regression model to predict the probability distribution of occurrences. Prior to running the regression analysis, the six original habitat predictors were converted to principal components (to reduce their dimensions and the multicollinearity effect) using the Hill-Smith ordination method [55] that deals with mixed variable types (i.e., quantitative and factor).

We used a generalised linear model (GLM) [56] for the regression analysis, assuming a binomial error in the response variable. Model residuals were then analysed by fitting a variogram to assess their level of spatial dependence (Figure 3). Model residuals exhibited no spatial dependence for six species (i.e., the squid, *Loligo vulgaris*; the stomatopod, *Squilla mantis*; the demersal fishes, *Merlucius merlucius*, *Mugil cephalus*, *Mullus barbatus*, and *Mullus surmuletus*; and the pelagic fish, *Trachurus trachurus*). For the remaining species (n = 21), we used logistic Regression Kriging (RK) models that explicitly incorporate spatial dependence into predictions (Figure 3). This method assumes that the model residuals have a spatial structure resulting from either ‘model’ factors such as incorrectly specified or inadequate predictor variables, or ‘real’ factors such as biotic processes that cause spatial patterns [57]. It combines the predictions from a regression model along with the resulting kriged residuals [58]. Specifically, the regression modelling was supplemented with the use of variograms to assess the level of spatial dependence among residuals (Figure 3). Regression residuals were then interpolated and added back to the regression estimate (see [58] for more details). Finally, to select the most parsimonious model for each of the selected species, we applied an automatic stepwise model selection using the Akaike Information Criterion [59].

#### Model accuracy

For every species, the predictive accuracy of the model was evaluated by a 10-fold cross validation [56]. The receiver operating characteristic curve method was then applied to derive the area under the curve (AUC) index [60] to measure the model’s performance.

The simulation of pseudo-absences may generate absences far from the environmental conditions of presences, which may artificially increases the rate of well-predicted absences and hence the AUC scores [24], [25], [61]. In addition, the AUC test statistic may not always reflect a model’s ability to prioritise areas in terms of their habitat suitability relative to alternative models (e.g., [61], [62]). Model assessment was therefore supplemented with the Point Biserial Coefficient (PBC) [20], [24], [63], the sensitivity (presences correctly predicted as presences), and the specificity metrics (absences correctly predicted as absences) [60]. The PBC was calculated as a Pearson’s correlation coefficient between the observation in the occurrence dataset (presence (1) or pseudoabsence (0)) and the prediction and therefore takes into account how far the prediction varies from the observation. An independent examination of the percentage of presence and absence errors was recommended by Lobo *et al* [61] to help in the model selection process according to the researcher’s goals, rather than the use of a synthetic measure such as the AUC. Predictions were further inspected visually and compared to plotted occurrence data in order to assess their plausibility.

Finally, we used the Pearson’s correlation coefficient to calculate the pairwise correlation between the final predictions maps derived from the 10 groups of pseudo-absences. This allowed us to assess the stability of predictions for each of the different weighted simulations of pseudo-absences.

Each data processing step was completed in *R*, drawing on code developed by Hengl *et al.* [37], and automating the calculation for several species simultaneously. For each modelled species, the regression models, 10-fold cross validation, and evaluation procedures were carried out for the 11 simulated groups of pseudo-absences.

### Conservation Planning Procedure

Probability of occurrence maps can be generated using the most accurate model selected for each species from the 11 pseudo-absences groups. Once these maps have been converted to presence-absence data, they can be used to identify areas that meet the conservation targets for selection of AAR deployment sites. These targets specify the inclusion of both favourable habitats for commercially-targeted species and areas of high density *P. oceanica* seagrass beds.

#### Threshold approaches and fuzzy modelling

The most common method used to convert probabilities of occurrence to presence-absence data is the use of an optimum probability threshold [64]. Different methods have been proposed to select a probability threshold [27]. Among these, the most widely used threshold optimisation criteria are:

1. *Sens = Spec* - The threshold where sensitivity equals specificity (i.e., where positive observations are equally as likely to be wrong as negative observations).
2. *Max (Sens*+*Spec)* - The threshold that maximises the sum of sensitivity and specificity (i.e., it minimises the mean error rates for both positive and negative observations). This threshold is equivalent to finding the point on the receiver operating characteristic curve whose tangent has a slope of one.
3. *MaxKappa* - The threshold that results in the maximum value of Kappa statistic.
4. *MaxPCC* - The threshold that results in the maximum percent of correctly classified observations.

**Figure 4:**
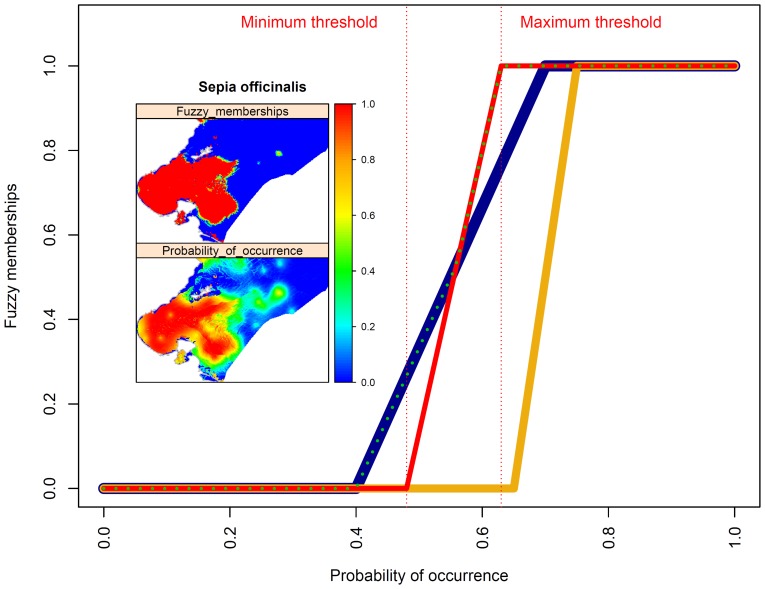
Threshold approaches and fuzzy modelling. An illustration of the fuzzification process performed using a fuzzy linear membership function. The red, orange, and blue curves represent the fuzzy membership sets of three different species. The dotted green line represents the fuzzy AND overlay outcomes. The inset maps display an example of the fuzzification process for the cuttlefish *(Sepia officinalis*) which corresponds with the red curve.

For each species, a probability threshold was determined using each of the four optimisation criteria outlined above. As these methods do not provide similar threshold values, the use of one method over another can influence conservation planning outcomes, e.g., modifying areas that are expected to be suitable for a given species [27]. To avoid the subjective selection of a particular threshold, predicted distributions of selected species were defined by fuzzy sets theory [65]. Fuzzy logic is useful in circumstances that involve uncertainty, imprecision, and vagueness by replacing the sharp boundary between the suitable and non-suitable classes with the concept of a degree of truth (membership). In the fuzzification process, crisp attribute values (probability of presence) are transformed linearly into a common suitability scale (0 to 1) using the fuzzy linear membership function. A membership value of 0 is assigned to the lowest probability threshold value (as calculated using the four criteria outlined above) and a value of 1 to the highest threshold value (see example in Figure 4). Each map cell was assigned a fuzzy membership value resulting from the fuzzy linear membership function.

#### Fuzzy overlay of conservation criteria

For each species, the favourability value of an area is defined as the degree of membership of that area to the fuzzy set of favourable areas for the species. Fuzzified inputs can be combined together to identify the most favourable area for the majority of commercial species by using a fuzzy operator. The fuzzy AND operator was applied to return the minimum of the fuzzy memberships from the fuzzy input maps. The result of this aggregation is a final fuzzy set expressing the site suitability for all key species (see example of the method in Figure 4). To identify areas with a high density of seagrass beds, a recent map showing seagrass recovery rates was obtained from the INSTM. This map was fuzzified using a fuzzy linear membership function that assigned a membership value of 0 for recovery rates less than 30% and a value of 1 for recovery rates greater than 60%. Finally, the maps that express the fuzzy memberships of site suitability for all key species and seagrass recovery rates were combined together using the fuzzy AND operator. For example, “IF the favourability value of an area for the species 1 IS high, AND the favourability value of an area for the species 2 IS high, etc…, AND the recovery rate of seagrass IS high THEN the area has high conservation criteria”.

## Results and Discussion

**Figure 5:**
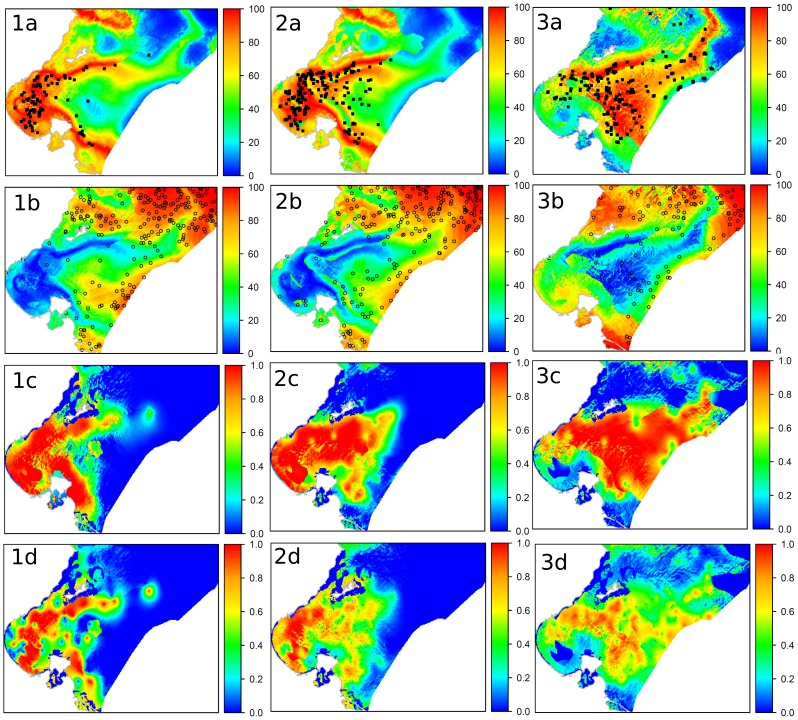
Spatial prediction maps in the Gulf of Gabes for three example species. (1) *Lithognathus mormyrus,* (2) *Penaeus kerathurus,* (3) *Pagellus erythrinus* (a) the habitat suitability index map with presence-only data; (b) the weighted map and the randomly-generated pseudo-absences using the Equation 1; (c) probabilities predicted using the binomial regression-kriging (RK) with a weighted selection of pseudo-absences; and (d) probabilities predicted using the binomial RK with a random selection of pseudo-absences.

The Monte-Carlo randomisation tests show that the ENFA marginality factor is highly significant for each modelled species (all p<0.001). This implies that the habitat occupied by the species modelled differ unequivocally from the average environmental conditions found in the broader study area. This indicates that a species-specific habitat selection process takes place. The ENFA also show that the potential distributions of species are much larger than their realised distributions, based on sampling locations (see examples of three species in Figure 5). In contrast, incorporating pseudo-absence data into logistic RK models results in predicted distributions that are closer to the realised distributions (Figure 5).

When the pseudo-absence data are randomly selected, the potential distribution of the species are less extensive in comparison with those obtained using a weighted selection. This difference can be explained by the fact that a random simulation can select absences between observed occurrences points, therefore generating pseudo-absences in favourable areas. This can subsequently lead to an underestimation in the realised distributions [25], [66].

**Figure 6:**
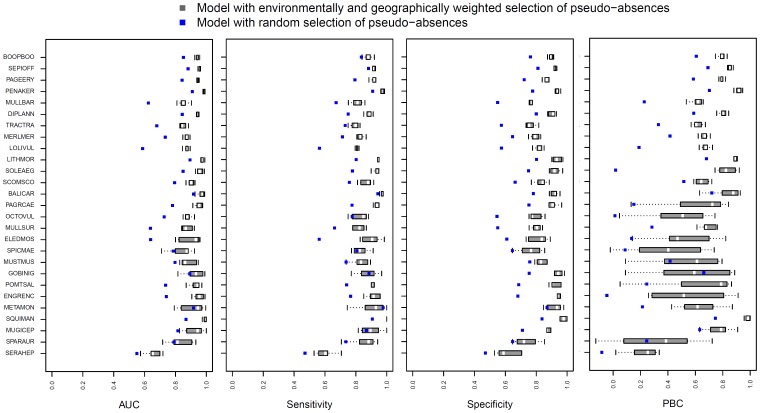
Distributions of the area under the curve (AUC), PBC, sensitivity and specificity values. Calculated AUC, PBC, sensitivity and specificity values for the 27 modelled species (species codes are listed in Table 1). Species are sorted (top to bottom) by decreasing number of occurrences (used to develop the models).

For each method generating pseudo-absences, the distribution of the AUC, PBC, the sensitivity and the specificity metrics is shown in Figure 6. For each species, model accuracy differs according to the method used to generate pseudo-absences. All the models based on the environmentally- and geographically-weighted method achieved a high level of accuracy as indicated by the four measures of model accuracy (Mean±standard deviation: 0.9±0.07, 0.65±0.23, 0.87±0.08 and 0.85±0.09 respectively for the AUC, PBC, sensitivity and specificity) (Figure 6). These values were significantly higher (Wilcoxon rank test: p<0.001) than those obtained by models based on the random selection method (Mean±standard deviation: 0.78±0.1, 0.36±0.26, 0.77±0.11 and 0.69±0.1 for the AUC, PBC, sensitivity and specificity, respectively).

Since all the resulting SDMs are based on pseudo-absences, both specificity and AUC scores estimate the degree of accuracy of the absence information used in the model training process. Thus, a high specificity score only implies that most of the data considered as absence data are correctly predicted and does not imply a high performance in the prediction of the unknown true absences. However, the sensitivity and PBC values were higher when the weighted method of generating pseudo-absence was used, implying a high performance of this method in the predictions of the known true presences as compared with the random method.

Models based on the weighted method of generating pseudo-absence data provide significantly better results on average, which aligns with the results of Engler *et al.,* Chefaoui & Lobo and Hengl *et al.* [22], [25], [37]. It contrasts, however, with the findings of Wisz & Guisan and Barbet-Massin *et al.* [19], [26]. Using virtual species, these authors found that randomly-selected pseudo-absences yielded the most reliable species distribution models. However, this may be explained by the fact that both studies used a large number of pseudo-absences (e.g., 10.000 data points), whereas this study used the same number of simulated pseudo-absences as the number of occurrences. Currently, there is no consensus on the number of pseudo-absences that are required to optimise model predictions. Some authors suggest a ratio of 10∶1 (e.g., [25], [49]) while others recommend using large numbers of pseudo-absences when they are randomly selected (e.g., [20], [26]). Intuitively, it makes sense to generate an equal number of pseudo-absence data points as occurrence data points [58], [67] to avoid the bias caused by a presence-absence ratio that is too low [68], [69]. Indeed this was the outcome in McPherson *et al.* [70]who found SDMs had the best predictive accuracy when prevalence values (the proportion of data points representing a species’ presence) were around 0.5. Therefore, several authors recommend resampling the training data to balance presence and absence data points [70], [71].

**Figure 7:**
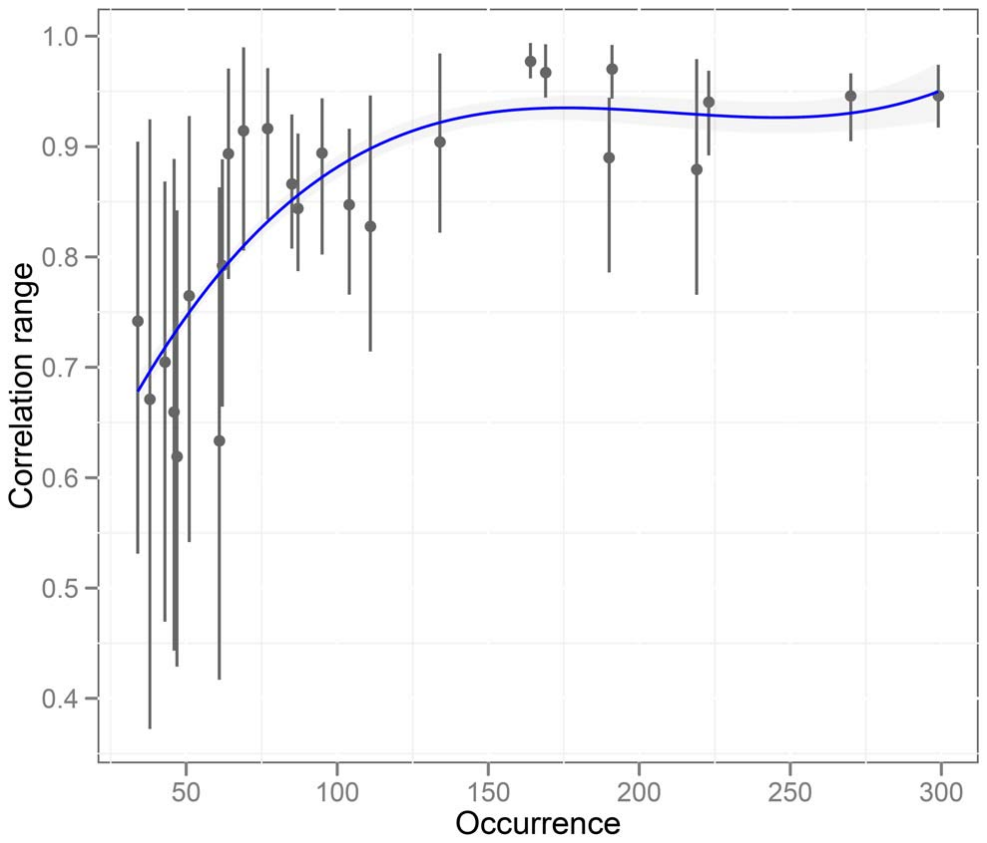
Stability of predictions. The range of Pearson’s pairwise correlation coefficients between the final prediction maps according to the number of occurrences used to develop the models (red line: smooth curve fitted by Loess function).

For each species, the range of pairwise correlations between the final habitat prediction maps (derived from each of the 10 groups of pseudo-absences) varied according to the number of occurrences used to develop the models (Figure 7). The lowest correlation occurs when the number of occurrences is less than 61. The array of evaluation measures, based on all the replicated runs, does not show a clear trend in relation to the number of occurrences used to fit the models. Even with a small number of occurrences, values of AUC and PBC indicate excellent predictive accuracy of the models. However, large fluctuations in the predictive accuracy and the models’ subsequent predictions are observed when the models are based on a low number of occurrences. Indeed, the selection of pseudo-absences can induce variability in model predictions when several runs are made with a small set of occurrence data, each run having its own dataset for calibration.

Williams [72] found that the predictive ability of some ecological modelling approaches varies with a species’ detectability. While presence-absence approaches generally have higher predictive abilities for species with a high detectability, they do not perform as well as presence-only approaches when detectability is low. However, for some species such as the pelagic fish species, *Trachurus trachurus*, the fitted model is found to be moderately accurate though it is based on a relatively high number of data points (338 presences/pseudo-absences). This result may be related to the fact that the selected predictors (e.g., depth, seafloor type) have greater ecological significance to model the distribution of demersal and benthic species. [66]. This implies that the performance of SDMs strongly depends on the environmental predictors, which in turn depend of the type of organism that is studied [62]. In addition, the realism and the robustness of models may have been influenced by our automatic variable selection procedure [73]. Indeed, ecologically important variables may have been excluded from the stepwise models and/or non-meaningful variables may have been incorporated into models. This was particularly the case for some of the studied pelagic species (*Trachurus trachurus, Spicara maena, Scomber scombrus*). These species were therefore excluded from the conservation planning procedure.

Overall, our results suggest that simulating pseudo-absences with an environmentally and geographically weighted method rather than a purely heuristic approach enhances the accuracy of predictions. This method provides a robust result when a relatively large number of occurrence data points with good spatial coverage are used. The RK method also shows great potential as an approach to incorporate spatial dependence in SDMs, by combining information on species-habitat relationships (i.e., through the deterministic model) and error components. We believe that the combined ENFA and RK method has several advantages when applied to trawl survey data, especially when addressing their imperfect ability to detect a species. Furthermore, this method applies both the spatial auto-correlation structure and the trend component of the spatial variation to make spatial predictions of species’ distributions. This method can be applied to other areas where survey data are available, such as the Medits (International bottom trawl surveys in the Mediterranean) dataset.

The second purpose of this study is to identify the areas required to meet the conservation targets of AARs based on probabilistic predicted maps. In this study we propose a novel method to develop these maps that uses fuzzy sets to address the uncertainty associated with the selection of probability threshold optimisation criteria. Fuzzy logic is interested in capturing partial truths, that is, how to reason about things that are not wholly true or false; while probability is concerned with making predictions about events based on a partial state of knowledge [65]. The fuzzy sets theory is used to transform the probability of presence into a membership degree using not only a single threshold value but several values obtained with different cut-off threshold optimization criteria.

Of the 27 species modelled in this study, an excellent or high predictive accuracy is only found for 12 benthic and demersal species. It was these species that were selected for the fuzzy overlay of conservation criteria. Of these, the 2004–2005 stock assessment results for the Gulf of Gabes report that *Solea aegyptiaca, Octopus vulgaris*, and *Sepia officinalis* were fully exploited (Othman Jarboui, personal communication: INSTM) and *Mullus barbatus, Pagrus caeruleostictis,* and *Pagellus erythrinus* were overexploited.

**Figure 8:**
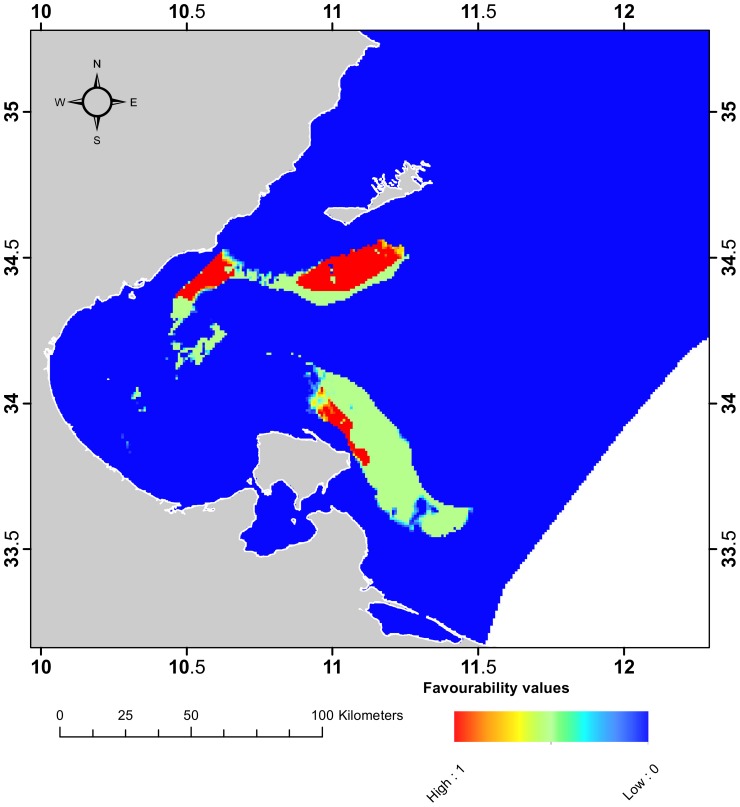
Map of favourability values for the deployment of AARs.

The resultant fuzzy overlay map (Figure 8) highlights three main areas that meet the conservation criterion to a high level. The largest area is located south of Kerkennah Island while the remaining two areas are in the coastal region off Mahres Harbour and north of Jerba Island. As well as meeting the AAR conservation criteria, the Kerkennah Island area has already been reported as a biodiversity hotspot for megabenthic fauna [74] and is currently proposed as a potential MPA [75]. The area north of Jerba Island was also proposed as suitable for MPA establishment by Ben Mustapha & Afli [75]. In addition to its dense seagrass beds, the area is also characterised by coralligenous assemblages and is a recognised nursery site for several juvenile of commercially-targeted species [75]. These expert opinions corroborate our findings and confirm the relevance of the established methodology for the selection of AAR deployment sites.

## Conclusions

The comparison between the two pseudo-absence data generation methods reveals that the environmentally- and geographically-weighted method has significant potential to reduce prediction errors in SDMs. When confirmed absence data are not available, we recommend using the method proposed by Hengl *et al.* [37], which combines ENFA and RK predictions to deal with an imperfect ability to detect a species and incorporate spatial dependence in predictions. This study proposes a novel method to developing predicted maps that uses fuzzy sets to address the uncertainty associated with selecting probability threshold criteria. In the context of conservation planning, we illustrate the advantages of this novel method, using it to identify areas within the Gulf of Gabes that meet the conservation targets for AARs. Three key areas that met the conservation criteria to a high level were identified, and these areas are recommended as deployment sites for AARs. The location and spatial arrangement of reef units must be carefully planned. Reef characteristics such as the number of modules, the distance between modules as a function of trawl parameters, and the weight of modules as a function of exposure to currents should all be informed by scientific studies. Additional factors that should be considered in the deployment of AARs are economic costs and socio-economic effects.

The areas with a nonzero favourability value for AAR deployment cover 1578 km^2^ and encompass 30% of seagrass beds in good condition. However, these areas represent biodiversity targets without regard to cost. In this context this study can contribute to effective conservation planning in a broader prioritization process, which must be optimized by accounting for the cost of conservation. For instance, future works should focus on systematic conservation planning [76], [77] that attempts to solve a cost-effectiveness problem (i.e. how to achieve the most conservation given limited resources). This is particularly important in the Gulf of Gabes where the diverse stakeholder group often holds conflicting values and opinions (e.g., conflicts between professional and local artisanal fishers).

## Acknowledgments

The authors would like to acknowledge the National Institute of Marine Sciences and Technologies (INSTM, Tunisia) that contributed unpublished data. We are very grateful to Jane Alpine for English editing. We also thank the two anonymous referees for their helpful comments on the manuscript.
